# Correlation between chronological age and skeletal age using cvmi and modified MP3 methods

**DOI:** 10.6026/973206300161045

**Published:** 2020-12-31

**Authors:** Reshma Mohan, Ravindra Kumar Jain, Nivethigaa Balakrishnan

**Affiliations:** 1 Saveetha Dental College, Saveetha Institute of Medical and Technical Sciences, Saveetha University, Chennai 77, India

**Keywords:** Maturity indicators, growth status, age prediction

## Abstract

Chronological age conveys only a rough approximation of the maturational status of a person whereas skeletal maturity indicators give a more accurate estimation. Therefore, it is of interest to document the correlation between chronological and skeletal age using CVMI and modified MP3 methods. A total of 39 subjects between the age ranges of 9-16 years were selected for this study. Pre-treatment lateral cephalograms and hand-wrist radiographs of the subjects were used. The skeletal age was analyzed by the Cervical Vertebrae Maturity Index (CVMI) and modified MP3 methods. The data was analyzed with SPSS software version 23.00. Kendall's Tau correlation test was performed to estimate the correlation between chronological age and skeletal age among the subjects and a linear regression test was also performed. Positive correlation was found between chronological age and skeletal age assessed by CVMI method (r= 0.398) and modified MP3 method (r=0.382) with p value >0.003. Thus it can be concluded that there was a positive correlation between chronological age and skeletal age among all the subjects.

## Background

Growth and Development of every individual is influenced by various factors like genetic, racial, nutritional, hormonal and climatic conditions [1,2]. According to Stewart RE and Barber TK [1] the age of an individual can be determined by various methods like chronological age, biological age, morphological age, skeletal age, dental age, circumpubertal age, morphological age, behavioural age, mental age and self-concept age. Chronological age is most easily determined developmental age obtained from the child's date of birth [2]. Since chronological age is not an accurate indicator of development of an individual, skeletal maturity indicators are preferred [3]. Skeletal age is determined by stages of ossification of the middle phalanx of the left hand third finger (MP3) as proposed by Hagg U and Taranger J [3]. It can also be done by cervical vertebral maturation method by Baccetti et al. [4]. Radiographic cephalometry, being an important investigative tool available to clinicians and researchers is used for a wide variety of diagnostic and comparative procedures and its potential for research purpose seems limitless [4]. The CVM method can be applied to determine the optimal treatment time in orthodontics to eliminate an extra hand-wrist radiograph [5]. Fishman L.S proposed the use of ossification centres seen in hand-wrist radiographs and cervical vertebrae in lateral cephalogram [6]. However its usage in pediatric patients is minimal due to reasons such as radiation exposure, high cost, etc [6]. To minimize the radiation exposure, the ossification of distal phalanx of the first digit was used by Godo et al, as an indicator to know the growth potential of that individual [7]. Abdel Khader HM [8] used MP3 stages for assessing skeletal maturity and later stated various advantages of digital radiography compared to that of conventional radiography like lower exposure time than conventional films and elimination of the darkroom procedures in addition to more clarity of the digital images.This method also fulfills the principle of As Low As Reasonably Achievable (ALARA) [9]. The MP3 method has many advantages over others such as low radiation exposure, high correlation with CVMI method, no superimposition of bone or variation in posture and no need for special X-ray equipment [2]. Therefore, it is of interest to document the correlation between chronological and skeletal age using CVMI and modified MP3 methods.

## Materials and methodology:

A total of 39 subjects were selected for this study. The data was retrieved from patient records provided by Saveetha Dental College, Chennai, India from June 2019 to March 2020. Two radiographs were obtained from the records of each patient - one lateral cephalogram for CVMI and hand-wrist radiograph for MP3 method. Prior to the start of the study, ethical approval was obtained from the Scientific Review Board of SIMATS with the following ethical approval number - SDC/SIHEC/2020/DIASDATA/0619-0320.

The inclusion criteria included:

[1] Age between 9 - 16 years 

[2] No prior orthodontic treatment

[3] Absence of developmental anomalies and craniofacial deformities

The skeletal age was calculated by modified MP3 method and by CVM method. The chronological age was calculated from the child's date of birth and subtracting it from the date obtained from the radiograph. All the values obtained were tabulated in Excel Spreadsheet and the radiographs were saved in Google Slideshare. SPSS software was used for statistical calculations. Kendall's Tau correlation test and linear regression tests were performed.

## Results and Discussion:

Kendall's Tau correlation test estimated the correlation between the chronological age and skeletal age among the study population (Table 1 - see PDF). Linear regression test was also performed in this study (Table 2 - see PDF). A positive correlation was found between chronological age and skeletal age as assessed by CVMI method (r=0.398) and modified MP3 method (r=0.382). The P value was 0.003 indicating that the correlation was statistically significant. Hence, a linear regression test was also performed. The linear equation derived from the regression test was, CVMI = 0.59x CA - 2.063 (Figure 1 - see PDF) and MP3 = 0.563 x CA - 1.764 (Figure 2 - see PDF). Previously our team had conducted numerous clinical trials [10-16], lab animal studies [17-21] and in - vitro studies [22-24] over the past 5 years. Now we are focussing on epidemiological surveys. The idea for this survey stemmed from the current interest in our community. Evaluation of age and maturational status will have a considerable impact on diagnosis, treatment planning and eventually the outcome of the treatment. This in turn could help in early correction of any skeletal or dental discrepancies in children. In the present study, a positive correlation was found between chronological age and skeletal age assessed by CVMI method (r= 0.398) and modified MP3 method (r=0.382) with p value <0.003. (Table 1 - see PDF) Similar results were reported by Madhulikha Macha et al. [2], Sachan et al. [25] and Vallejo-Bolanos et al. [26] where there is a positive correlation between chronological age, dental age and skeletal age. In a study conducted by Hessa Abdulla in the Southern Chinese population, there is a high correlation between CVMI and hand-wrist method [5]. This high correlation may be due to confining the correlation within the circumpubertal period. It may also be due to the ethnic characteristics of the southern Chinese population. In the present study, both methods of skeletal maturity measurements were sensitive and precise in evaluating the maturity changes. The correlation obtained between the CVM method and the Hand Wrist method applies to the South Indian population in this study. In contrast to the findings of this study, Patil.N et al. [27] conducted a study among the central Indian population correlating between chronological age, cervical vertebral maturation and Fishman's Skeletal Maturity Indicator. It was found that even though there is a high correlation between CVM method and HWM, there was no correlation between chronological age and skeletal maturity indicators. Similar results were found in a study conducted by Kiran S et al. [28] where they correlated chronological age, cervical vertebral maturation and skeletal maturity indicators in the Lucknow population. The limitation of this study includes a small sample size and also the study focuses only a certain group of population residing in Chennai city of Tamil Nadu state in India. Hence, further research is required on a larger sample with new population groups.

## Conclusion:

From the present study it can be concluded that there is a significant correlation between chronological and skeletal age and hence chronological age is a good indicator of skeletal development.

## Clinical significance:

Skeletal maturity assessment is very important in orthodontic diagnosis and treatment planning since many treatment interventions require growth modifications.
